# Kinetics of NiO and NiCl_2_ Hydrogen Reduction as Precursors and Properties of Produced Ni/Al_2_O_3_ and Ni-Pd/Al_2_O_3_ Catalysts

**DOI:** 10.1155/2015/601970

**Published:** 2015-02-18

**Authors:** Miroslav Sokić, Željko Kamberović, Vesna Nikolić, Branislav Marković, Marija Korać, Zoran Anđić, Milorad Gavrilovski

**Affiliations:** ^1^Institute for Technology of Nuclear and Other Mineral Raw Materials, 86 Bulevar Franš d'Eperea Street, 11000 Belgrade, Serbia; ^2^Faculty of Technology and Metallurgy, University of Belgrade, 4 Karnegijeva Street, 11120 Belgrade, Serbia; ^3^Innovation Center of the Faculty of Technology and Metallurgy, University of Belgrade, 4 Karnegijeva Street, 11120 Belgrade, Serbia; ^4^Innovation Center of the Faculty of Chemistry, University of Belgrade, 12-16 Studentski Trg Street, 11000 Belgrade, Serbia

## Abstract

The objects of this investigation were the comparative kinetic analysis of the NiO and NiCl_2_ reduction by hydrogen during an induction period and elimination of the calcination during the synthesis of Ni/Al_2_O_3_ catalysts. The effect of temperature and time on NiO and NiCl_2_ reduction degrees was studied. Avrami I equation was selected as the most favorable kinetic model and used to determine activation energy of the NiO and NiCl_2_ reduction for the investigated temperature range (623–923 K) and time intervals (1–5 minutes). The investigation enabled reaching conclusions about the reaction ability and rate of the reduction processes. Afterward, Ni/Al_2_O_3_ catalysts were obtained by using oxide and chloride precursor for Ni. The catalysts were supported on alumina-based foam and prepared via aerosol route. Properties of the samples before and after low-temperature hydrogen reduction (633 K) were compared. Obtained results indicated that the synthesis of Ni/Al_2_O_3_ catalysts can be more efficient if chloride precursor for Ni is directly reduced by hydrogen during the synthesis process, without the calcination step. In addition, Ni-Pd/Al_2_O_3_ catalysts with different metal content were prepared by using chloride precursors. Lower reduction temperature was utilized and the chlorides were almost completely reduced at 533 K.

## 1. Introduction

Nowadays, nickel is one of the most technologically important metals due to its physical and chemical properties. It is widely used as the alloying element, particularly in demanding corrosion-resistant applications [1–3]. The production of fine Ni powders, which are used in electronics industry and for the polycomponent catalysts manufacturing, is a great challenge in the field of new materials [4–6]. A widely used method for Ni powder production is reduction of NiO by hydrogen:

(1) $$\begin{array}{c} \text{NiO}\left(\text{s}\right)+{\text{H}}_{2}\left(\text{g}\right)=\text{Ni}\;\left(\text{s}\right)+{\text{H}}_{2}\text{O}\left(\text{g}\right) \end{array}$$

Two main characteristics in the kinetics of metal oxide reduction are the existence of an induction period and the possibility for autocatalysis [7, 8]. On the basis of both theoretical and experimental analyses of the NiO reduction by hydrogen, Delmon [9] determined number of potential Ni nuclei and the other parameters which confirmed the law of the new phase nuclei formation. Eliminating the influence of the diffusion controlled stage, Delmon reduced the spherical NiO particles (diameter from 0.7 to 0.8 *μ*m). The reduction was carried out in a temperature range from 406 to 498 K, with both the homogeneous and the heterogeneous nucleation of Ni on the added foreign particles. The foreign particles, as the Ni crystallization nuclei, caused positive effect on the NiO reduction rate. The induction period of reduction process, which occurred with the homogeneous nucleation, lasted 2.2 minutes, while in the process occurring with addition of foreign nuclei, it lasted 1.6 minutes. Richardson et al. [10] studied mechanism of the reduction of porous bulk NiO at elevated temperatures (448–573 K). Their results show a direct NiO → Ni transformation without accumulation of any intermediate phase. Without addition of water to the reducing gas (H_2_), reduction had the following stages: (1) an induction period, where the initial NiO reduction occurred and Ni clusters were formed, (2) increase of the reduction rate with the enlargement of Ni clusters, and (3) a pseudo-first-order (excess H_2_) process, where NiO disappeared, Ni was formed instead, and the reduction slowed at a fractional conversion of about 0.8 [10]. A number of research groups greatly contributed to investigation of kinetics and mechanism of the NiO reduction by hydrogen. Activation energy values for the NiO reduction were 20 and 19 kJ/mol for powder and pellet, respectively [11], and 90 kJ/mol for Goro NiO [12]. Plascencia and Utigard [7] determined activation energy for the reduction of two commercial nickel oxides. The values were 32 and 30 kJ/mol for Tokyo and Sinter 75 sample, respectively. Janković [13] reduced NiO prepared by sol-gel method. Experimental results were compared with 15 theoretical reaction models. He proposed sequential-reaction mechanism and average apparent activation energy value was 101 kJ/mol. Considering other metal oxides, Piotrowski et al. [14] obtained activation energy values of 28 and 123 kJ/mol when Fe_2_O_3_ was reduced by H_2_ and CO, respectively. According to that research group, the processes were initially chemically controlled [14]. According to Go et al. [15], the water splitting reaction with Fe_2_O_3_, ZnFe_2_O_4_, and MnFe_2_O_4_ was controlled by the product-layer diffusion. Activation energy values ranged from 57 to 110 kJ/mol.

Another method for the synthesis of Ni powder is reduction of NiCl_2_ by hydrogen:

(2) $$\begin{array}{c} {\text{NiCl}}_{2}\left(\text{s}\right)+{\text{H}}_{2}\left(\text{g}\right)=\text{Ni}\left(\text{s}\right)+2\text{HCl}\left(\text{g}\right) \end{array}$$

In comparison to the NiO hydrogen reduction, the kinetics of the NiCl_2_ reduction by the same reducing agent has been the subject of a smaller number of studies. Suh et al. [16] investigated the kinetics of gas phase reduction of NiCl_2_. The aim was to produce Ni particles in hydrogen atmosphere using a tubular furnace reactor. They obtained the activation energy value of 104 kJ/mol. Stopić et al. [17] conducted kinetic analysis of NiCl_2_ reduction, with 0.1 wt% of Pd, Cu, or Ni and without additives. The activation energy of NiCl_2_ reduction, at temperatures from 573 to 823 K, was 54 kJ/mol. The presence of additives caused a decrease in the activation energy at lower temperatures (533 to 653 K): 33 kJ/mol for Ni(Pd); 50 kJ/mol for Ni(Cu) and Ni(Ni). The influence of Pd was explained by dissociation and hydrogen spillover, where reactive molecular hydrogen was formed and intensified the reduction process. Good solubility of Cu in Ni decreased energy bonds in the Ni lattice and added Ni(HCOO)_2_ formed artificial Ni nuclei. Effect of Pd to a decrease in particle size and improved uniformity of particle shape was the most dominant. Stopić et al. [18] synthesized fine nanosized Ni powder by ultrasonic spray and hydrogen reduction pyrolysis. Addition of Pd and Cu enabled complete NiCl_2_ reduction at 1173 K in dynamic conditions. When Pd was added, ideally spherical nonagglomerated Ni particles were obtained. The presented method was proven to be efficient for the production of fine nanocrystalline Fe-Ni alloy particles from corresponding metal chlorides [19]. Pérez-Hernández et al. [20] successfully produced Ni-based catalysts by impregnation and they used NiCl_2_ as a precursor for Ni. That preparation method included calcination at 773 K for 2 h in air and reduction by hydrogen for 2 h at 773 K [20]. However, the calcination may not be necessary during the synthesis of catalysts. In the earlier work of authors [21], addition of Pd, Cu, and Fe positively influenced the reduction process of NiCl_2_ in hydrogen atmosphere, at low temperatures (533 to 653 K). The highest reduction degree of 58 wt% was achieved with Pd addition at 653 K for 24 minutes. It was concluded that obtained results can particularly be significant for the synthesis of Ni-based catalysts. Reduction of chloride precursors can enable economic and technological benefits in the catalysts production, by elimination of the calcination step and reduction at low temperatures. According to Juan-Juan et al. [22], the calcination (773 K or 973 K for 12 h) can be avoided. Ni/Al_2_O_3_ catalysts prepared by impregnation and obtained by the direct hydrogen reduction of nickel salt at 773 K or 973 K for 2 h had good performance in reforming of CH_4_ with CO_2_. These authors noted that the calcination caused formation of hardly reducible NiAl_2_O_4_ phase in the catalysts [22]. It is known that modification with other active components, especially noble metals (Pd, Pt), improves catalytic properties of Ni-based catalysts [23, 24].

Catalytic activity can be enhanced if active components are supported on reticulated ceramic foams [25]. Using of alumina-based foams, synthesized as described in the previous research of authors [26], can contribute to energy savings in the complete process of catalyst manufacturing. The foams with improved mechanical properties were prepared by polymer replication. In comparison to the current synthesis processes, lower temperature was utilized during the sintering step (1673 K) [26]. Authors developed synthesis method for Ni-based catalysts supported on alumina-based foams in the earlier work [27]. That investigation included dependence of the catalyst properties from synthesis method and the addition of Pd, Cu, and Fe. The foams were impregnated with ultrasonically aerosolized solutions of metal chlorides. Oxides and chlorides on the foam surface were obtained by calcination and drying, respectively. Then, the samples were reduced by hydrogen at low temperatures (533 and 633 K). Ni/Al_2_O_3_ catalyst prepared by using chloride precursor had Ni coating without agglomerates and cracks, while those obtained from oxide precursor had residual agglomerates. All of the samples with chlorides reached higher reduction degree than those with oxides. The catalyst with 0.1 wt% of Pd, prepared from chlorides, was nearly completely reduced at 533 K [27].

This paper aims to compare kinetics of NiO and NiCl_2_ reduction by hydrogen during an induction period and to consider the possibility of direct reduction of chloride precursor for Ni during the catalysts synthesis. First, kinetic analysis was made in order to reach conclusions about reduction mechanism. By testing kinetic equations for solid state reactions [28], it was concluded that the experimental results were best fitted by Avrami I equation. Activation energy of NiO and NiCl_2_ reduction was determined from slopes of corresponding Arrhenius plots and by using Arrhenius equation. Afterwards, Ni/Al_2_O_3_ catalysts with 20 wt% Ni loading, supported on alumina-based foam, were prepared by using aerosol route, as previously described [27]. Ni coating was obtained from oxide and chloride precursor. For comparison purposes, the following catalysts were also synthesized: Ni/Al_2_O_3_ with 5 wt% Ni loading and Ni-Pd/Al_2_O_3_ with 5 wt% and 20 wt% Ni loading. For synthesis of these catalysts, only chloride precursors for Ni and Pd were used. Properties of the samples before and after hydrogen reduction were examined. Elimination of the calcination step and the reduction at low temperature were proposed.

## 2. Materials and Methods

For the reduction of NiO and NiCl_2_, the used materials included NiO powder and NiCl_2_ × 6H_2_O (MERCK, pro analysis). The NiCl_2_ powder was prepared by dehydration of NiCl_2_ × 6H_2_O for 1 h at 473 K and grinding in a mortar. The experimental investigations of NiO and NiCl_2_ reduction were conducted in a tubular quartz reactor set in an electric resistance furnace. Silica vessel carriers contained 10 g of each powder. The samples were reduced in static conditions, from 1 to 5 minutes at temperatures from 623 to 923 K (hydrogen flow rate 18 L/h).

**Figure 1:**
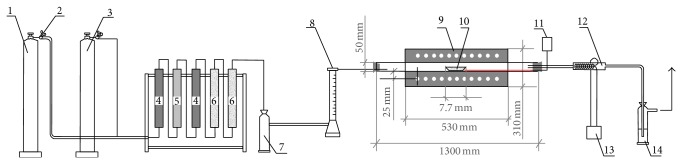
The apparatus used for reduction of NiO and NiCl_2_ in static conditions: 1: high-pressure hydrogen bottle, 2: reducing valve, 3: high pressure nitrogen bottle, 4: silica gel column, 5: copper powder column, 6: calcium-chloride column, 7: wash bottle with H_2_SO_4_, 8: rotameter, 9: electric resistance furnace with quartz tube, 10: silica sample carrier, 11: thermocouple, 12: water-cooled condenser, 13: water-cooling system, and 14: wash bottle.

Apparatus for the reduction experiments is illustrated in Figure 1 and presented in the previous research of authors [21]. Hydrogen was supplied from the high-pressure container linked to the columns system for purification (the silica gel, the calcium chloride, and the copper powder columns) through the wash bottle with H_2_SO_4_. The electric furnace with special construction provided a simple furnace opening. Automatic measuring and regulation of temperature during the reduction process was enabled. The reaction tube and silica carrier for NiO and NiCl_2_ samples ensured high temperatures operation.

A flow of nitrogen was injected into the reaction area in order to remove the residual oxygen and attain a neutral atmosphere. After the nitrogen inlet was stopped, hydrogen was introduced through the wash bottle (7) and the rotameter (8) into the silica tube containing the silica vessel (10) with the NiO or NiCl_2_ sample. Then, the silica tube was put into the electric furnace, previously heated to the selected temperature. The reaction time was measured from that moment. During the reduction, gaseous products were led away through the water-cooled condenser (12) and the wash bottle (14) to the gas spout.

After completion of the desired reduction time, the silica carrier was taken away from the tube and cooled down in a desiccator to room temperature. The reduction degrees were determined on the basis of the sample mass losing during the reduction.

The Avrami (I) equation was applied to data obtained for time-temperature dependence of reduction degrees. Then, activation energy values were determined from slopes of Arrhenius plots and by using the Arrhenius equation.

Reduction of NiO and NiCl_2_ was carried out at temperatures from 623 to 923 K in order to investigate the reaction ability and rate of the reduction processes at low temperatures. Obtained results were the basis for further research, where Ni/Al_2_O_3_ catalysts were synthesized by using oxide and chloride precursor for Ni. The aim was to select precursor that offers the following two possibilities during the catalyst synthesis: elimination of the calcination step and reduction of Ni/Al_2_O_3_ catalysts at low temperature (633 K).

For the synthesis of Ni/Al_2_O_3_ catalysts, the following materials were used: NiCl_2_ × 6H_2_O (MERCK, pro analysis) and alumina-based foam that was produced as described in our earlier work [26]. The catalysts with 20 wt% of Ni were obtained according to previously presented method [27]. First, aqueous solution of NiCl_2_ with metallic ion concentration of 0.15 mol/L was prepared. Then, the foams were placed in a tubular quartz reactor and impregnated with ultrasonically aerosolized chloride solution by using two different procedures. In the first procedure, impregnation at 773 K and calcination at 773 K were carried out for 1 h, which resulted in formation of oxide precursor for Ni on the foam surface. In the second procedure, chloride precursor was formed by impregnation at 473 K and drying at that temperature for 1 h. After the calcination and drying, the samples were reduced by hydrogen at 633 K for 1.5 h (static conditions, H_2_ flow rate: 20 L/h). Apparatus for the impregnation experiments was presented in the previous research of authors [27]. Subsequently, the following catalysts were prepared: Ni/Al_2_O_3_ and Ni-Pd/Al_2_O_3_ with 5 wt% Ni loading and Ni-Pd/Al_2_O_3_ with 20 wt% Ni loading. With respect to Ni, modified catalysts contained 0.1 wt% of added Pd. Those samples were synthesized according to the second procedure in order to obtain chlorides on the foam surface. The same materials were used for the synthesis of all the samples, except that the additional material was PdCl_2_ (MERCK, pro analysis). Lower amount of chloride solutions (0.15 mol/L metallic ion concentration) was used to decrease metal content. After the drying, the samples were reduced in hydrogen flow at 533 and 633 K for 1.5 h (static conditions, H_2_ flow rate: 20 L/h).

SEM analysis of the samples, before and after the reduction, was conducted in order to compare their properties.

## 3. Results and Discussion

**Figure 2:**
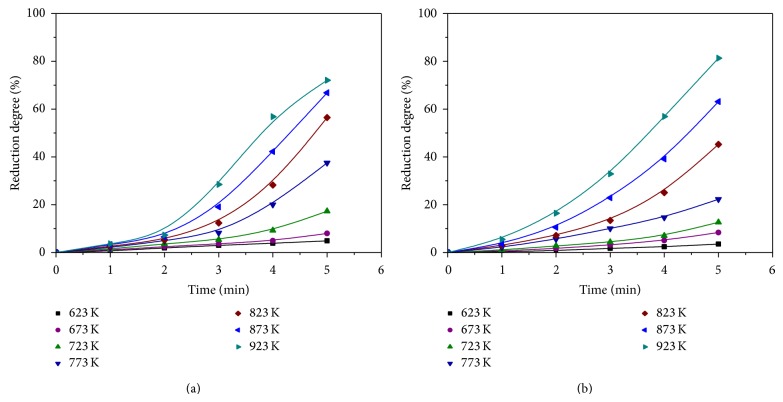
Effect of reduction temperature and time on the reduction degree of (a) NiO and (b) NiCl_2_.

The influence of reduction temperature and time on the NiO and NiCl_2_ reduction degrees is illustrated in Figure 2.

As expected, reduction degrees of NiO and NiCl_2_ increased with an increase in time and temperature. The peak reduction degree values reached 72 and 81 wt% for NiO and NiCl_2_, respectively, at 923 K for 5 minutes. Regarding the NiO reduction (Figure 2(a)), obtained results clearly show a pronounced induction period of 2 minutes, which decreases with an increase in temperature. In comparison with NiO, it is clearly visible that the induction period of NiCl_2_ was less pronounced and lasted about 1 minute (Figure 2(b)). This is the most probable explanation for slightly higher reduction degree of NiCl_2_, compared to NiO, at the same time and temperature. Induction period is an initiation of the reduction process, which occurs before actual reduction reaction is started. During induction period, crystallization centers of new phase (nuclei) start to develop and then begin to grow. After that, the reaction accelerates [7, 8]. In general, induction period lasts longer at lower reduction temperatures.

Kinetic analysis of the NiO and NiCl_2_ reduction by hydrogen was made on the basis of the experimental results shown in Figure 2, by using appropriate kinetic equations [28] (Table 1).

By testing these equations, it was concluded that the experimental results of the temperature influence on the NiO and NiCl_2_ reduction degrees were best fitted by Avrami I equation:

(3) $$\begin{array}{c} {\left[-\ln\left(1-\alpha \right)\right]}^{1/2}=kt, \end{array}$$

where *k* is a rate constant with unit of reciprocal time and *α* is reduction degree (wt%).

**Figure 3:**
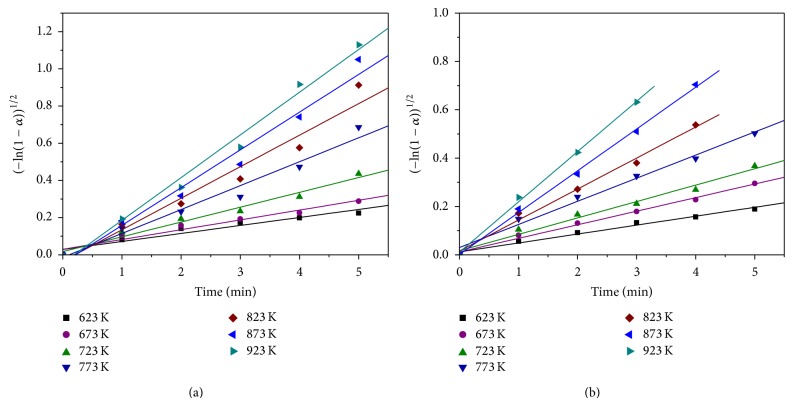
Linear correlation between [−ln⁡(1 − *α*)]^1/2^ and time for various temperatures: (a) NiO and (b) NiCl_2_.

Linearization of the experimental results by Avrami I equation is presented in Figure 3.

Equation (3) was developed assuming the cylindrical shape of particles, the accidental nucleation of new phase, and the spreading of the reaction front (surface, area) in the beginning stage of reaction. The excellent linear correlations between [−ln⁡(1 − *α*)]^1/2^ and time for various temperatures indicate that, during the induction period, the reduction of NiO and NiCl_2_ by hydrogen is controlled by chemical reaction.

The relationship between the basic kinetic parameters of a process (rate constant—*k* and activation energy—*E*_*a*_) is usually described by Arrhenius equation:

(4) $$\begin{array}{c} k=k_{0}\cdot e^{-E_{a}/RT}. \end{array}$$

**Figure 4:**
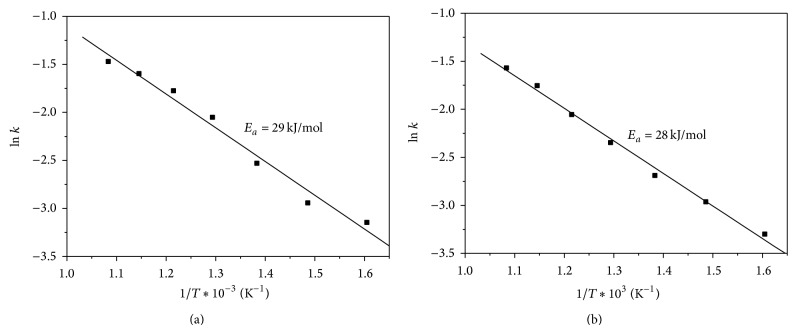
Arrhenius plot for the reduction of (a) NiO and (b) NiCl_2_ by hydrogen during the induction period.

The rate constant *k* for various temperatures was determined from the slope of the curves presented in Figure 3 and obtained values were used for the Arrhenius plots. The activation energy values for the NiO and NiCl_2_ reduction during the induction period were calculated from the slopes of the plots, using Arrhenius equation, as shown in Figure 4.

The activation energy values for the temperature interval from 623 to 923 K were 29 and 28 KJ/mol for NiO and NiCl_2_ reduction, respectively (Figures 4(a) and 4(b)). Although the obtained activation energies are not typical for the kinetic region, time intervals of the NiO and NiCl_2_ reduction were short.

Considering that the experimental results were best fitted by using Avrami I equation (3), it was concluded that the reduction processes were controlled by chemical reaction. Piotrowski et al. [14] calculated activation energy values of 28 and 123 kJ/mol for the reduction of Fe_2_O_3_ by H_2_ and CO, respectively. The research group has reported that the processes were initially chemically controlled. For the reduction with both reducing agents, the same mechanism was suggested. Go et al. [15] investigated the water splitting reaction with Fe_2_O_3_, ZnFe_2_O_4_, and MnFe_2_O_4_. The reaction was controlled by the product-layer diffusion and activation energy values varied from 57 to 110 kJ/mol.

In the present research, slightly lower activation energy of the NiCl_2_ reduction indicates that the NiO reduction rate is slower compared to NiCl_2_. In addition, obtained results show that NiO phase is more stable and less reducible than NiCl_2_.

After the comparative kinetic analysis, it was concluded that using of chloride precursor is promising for the synthesis of Ni powder and Ni-based catalysts, enabling lower energy consumption.

**Figure 5:**
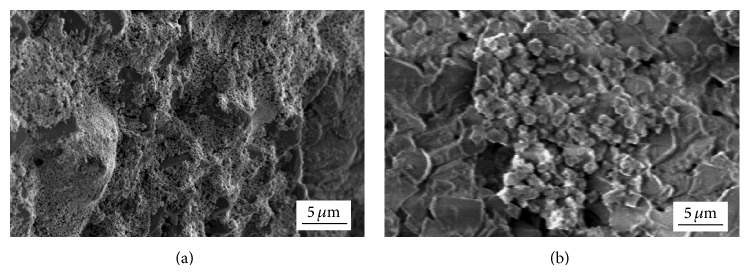
SEM micrographs of samples with 20 wt% Ni loading: (a) oxide precursor for Ni on the foam surface and (b) Ni on the foam surface, obtained after reduction of oxide precursor at 633 K.

In the further course of research, authors synthesized Ni/Al_2_O_3_ catalysts with 20 wt% of Ni. SEM micrographs of oxide precursor (after calcination at 773 K for 1 h) and Ni coating (after reduction at 633 K for 1.5 h) on the foam surface are presented in Figure 5.

**Figure 6:**
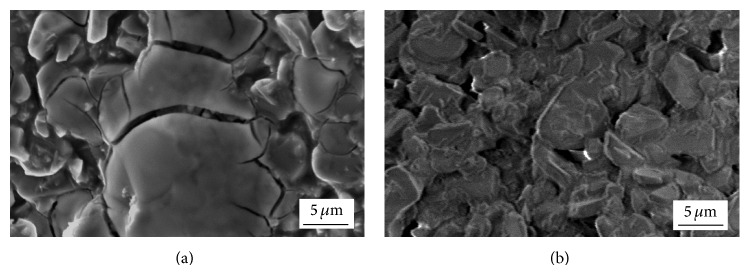
SEM micrographs of samples with 20 wt% Ni loading: (a) chloride precursor for Ni on the foam surface and (b) Ni on the foam surface, obtained after reduction of chloride precursor at 633 K.

Figure 6 shows SEM micrographs of chloride precursor (obtained by drying at 473 K for 1 h) and Ni coating (after reduction at 633 K for 1.5 h) on the foam surface.

The calcined sample, shown on Figure 5(a), had sponge-like agglomerates and uneven particle distribution. This morphology was most probably obtained because of the combined complex mechanisms of rapid water evaporation from the aerosol droplets, dehydration of NiCl_2_ × 6H_2_O, and oxidation of NiCl_2_ at 773 K. Due to cell morphology of the foam, its surface was not completely covered with oxide precursor. After the reduction at 633 K, a Ni coating completely covered the foam due to complex mechanism of mass transport. However, large residual sponge-like agglomerates were noted (Figure 5(b)). Calcination treatment increases stability of oxides, which is probable explanation for the residual agglomerates after the reduction. In contrast to that, SEM of the dried sample shows complete coverage of the foam surface with a chloride precursor layer (Figure 6(a)). For the synthesis of this sample, lower temperature was applied during the impregnation and drying. Evaporation of water from the aerosol droplets was slower, which resulted in this morphology of the chloride layer. The chloride layer had cracks and inhomogeneous thickness. The cracks appeared due to the evaporation of water from the aerosol and dehydration of NiCl_2_ × 6H_2_O at 473 K. The main reason for inhomogeneous distribution of chloride particles could be the foam cell morphology. Although the crust was cracked and had uneven thickness, relatively smooth Ni coating was formed after the reduction (Figure 6(b)). That coating covered the whole foam surface. In addition, cracks and agglomerates were not noted. The complete coverage of the foam with Ni coating and disappearance of cracks can be explained by complex mechanism of mass transport during the reduction. Mass transport took place when Ni atoms gained sufficiently high diffusion mobility at elevated temperature. Contacts between Ni particles were formed in cracks of the chloride layer and the growth of contact surfaces occurred. The morphologies of Ni coatings on the foam surface, obtained after the reduction of oxide and chloride precursors, indicate that the chloride phase has higher reducibility than the oxide phase. That is consistent with the comparative kinetic analysis results and it was confirmed in our earlier work [27]. Reduction degree of the sample obtained from chloride precursor (35.2 wt%) was approximately twice higher than that of the sample obtained from oxide (15.4 wt%) at 633 K [27]. It was concluded that the Ni/Al_2_O_3_ catalysts can be synthesized by the direct reduction of chloride precursor on the foam surface, without the calcination step.

The further course of research included preparation of Ni/Al_2_O_3_ and Ni-Pd/Al_2_O_3_ catalysts with 5 wt% Ni loading by using only chloride precursors for metals. These samples were synthesized in order to investigate influence of Ni loading and the addition of Pd on properties of the catalysts. For comparison purposes, Ni-Pd/Al_2_O_3_ catalyst with 20 wt% Ni loading was also prepared.

**Figure 7:**
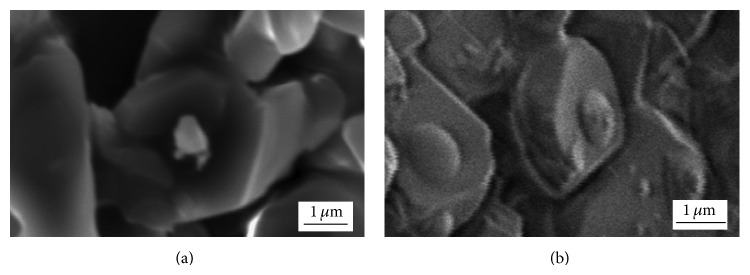
SEM micrographs of samples with 5 wt% Ni loading: (a) chloride precursor for Ni on the foam surface and (b) Ni on the foam surface, obtained after reduction of chloride precursor at 633 K.

SEM micrographs of chloride precursor (obtained by drying at 473 K for 1 h) and Ni particles (after reduction at 633 K for 1.5 h) on the foam surface are shown in Figure 7.

SEM analysis of the dried sample clearly showed presence of fine, submicron-sized, and relatively unevenly dispersed chloride particles on the foam surface (Figure 7(a)). Lower amount of the chloride solution and the foam cell morphology led to formation of this microstructure. After the reduction at 633 K, submicron-sized, isolated, and island-like Ni particles were obtained (Figure 7(b)). Reduction degree of Ni/Al_2_O_3_ sample with 5 wt% Ni loading significantly increased and it reached a value of 67.7 wt% at 633 K. Considering Ni/Al_2_O_3_ sample with 20 wt% Ni loading, 35.2 wt% of chloride precursor was reduced at the same temperature [27]. The morphology obtained with lower chloride content resulted in higher reduction degree because fine isolated chloride particles had good exposure to hydrogen stream.

High reducibility of the catalysts at low temperatures (533 and 633 K) is achieved by modification with only 0.1 wt% of Pd. Active Ni and Pd particles can be obtained by the direct reduction of the corresponding chlorides, supported on a ceramic support. These chlorides are nearly completely reduced at 533 K: reduction degree of Ni-Pd/Al_2_O_3_ sample with 20 wt% Ni loading, and 0.1 wt% of Pd reaches a value of 98.2 wt% [27]. In the present investigation, properties of synthesized Ni-Pd/Al_2_O_3_ catalysts with different metal loading were compared. Metals were obtained by reduction of corresponding chlorides and lower reduction temperature (533 K) was utilized.

**Figure 8:**
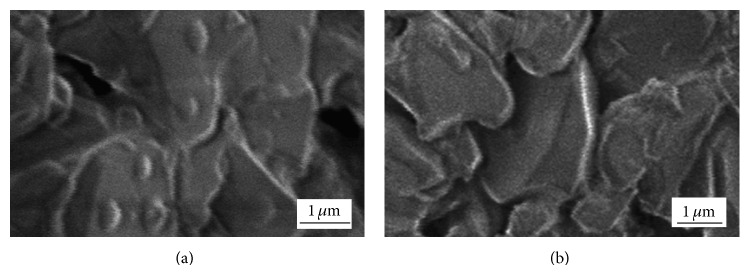
SEM micrographs of Ni-Pd on the foam surface, obtained after reduction of chloride precursors at 533 K, Ni loading: (a) 5 wt% and (b) 20 wt%.

SEM micrographs of Ni-Pd/Al_2_O_3_ catalysts with 5 wt% and 20 wt% Ni loading (after reduction at 533 K for 1.5 h) are presented in Figure 8.

After the SEM analysis of reduced Ni-Pd/Al_2_O_3_ sample with 5 wt% of Ni, fine, submicron-sized, and isolated island-like particles were noticed on the foam surface (Figure 8(a)). Higher Ni loading (20 wt%) resulted in formation of relatively smooth Ni-Pd coating over the whole foam surface (Figure 8(b)). Ni-Pd/Al_2_O_3_ catalysts (reduced at 533 K) and Ni/Al_2_O_3_ catalysts (reduced at 633 K) had similar morphologies for the same metal loading. Chloride precursors for Ni and Pd were almost completely reduced at 533 K. Reduction degree of Ni-Pd/Al_2_O_3_ with 5 wt% Ni loading reached a value of 99.4 wt%. This value was only slightly higher than the reduction degree of Ni-Pd/Al_2_O_3_ with 20 wt% Ni loading (98.2 wt%) [27]. Considering that similar reduction degrees were obtained for different metal loading, Ni-Pd/Al_2_O_3_ catalysts with 20 wt% of Ni could be more suitable for catalytic processes because the metal coating completely covers the foam surface.

Presented results indicate that the calcination step can successfully be eliminated during the production of Ni-based catalysts. In addition to low-temperature reduction of the catalysts, it is possible to achieve further energy savings by using alumina-based ceramic foams that are sintered at low temperatures.

## 4. Conclusions

Analysis and study of the experimental results showed that the NiO and NiCl_2_ reduction by hydrogen occurred in the temperature range of 623 to 923 K. The NiCl_2_ and NiO reduction degrees reached peak values of 81 and 72 wt%, respectively, at 923 K for 5 minutes. Considering both systems, the duration of the induction period decreased with the increase of temperature. Under the same reduction conditions, slightly higher NiCl_2_ reduction degree was obtained due to less pronounced induction period (about 1 minute).

The experimental results of the temperature influence on the reduction degrees were best fitted by Avrami I equation, which is applied when the rate of a process depends on the rate of chemical reaction and when the reaction front spreads until the maximum reaction rate is achieved. The activation energy values for the induction period of the NiO and NiCl_2_ reduction were 29 and 28 KJ/mol, respectively, in the temperature interval from 623 to 923 K. Obtained results indicated that the rate of NiO reduction was slower compared to NiCl_2_.

It was concluded that the calcination step could successfully be eliminated by direct reduction of chloride precursor in the manufacturing of Ni/Al_2_O_3_ catalysts. Ni coating, supported on alumina-based foam, was synthesized from oxide and chloride precursor. Higher reducibility of the chloride phase was confirmed after the examination of prepared Ni/Al_2_O_3_ catalysts with 20 wt% Ni loading. Reduction degree of chloride on the foam surface reached approximately twice higher value (35.2 wt%) than the oxide (15.4 wt%) at 633 K. In contrast to Ni coating obtained from oxide precursor, when chloride was reduced at 633 K, relatively smooth coating without residual agglomerates and cracks was formed. When Ni/Al_2_O_3_ catalyst with lower Ni loading (5 wt%) was obtained by using chloride precursor, significantly higher reduction degree (67.7 wt%) was achieved at 633 K. Submicron-sized isolated Ni particles were formed on the foam surface. Additional energy savings in the production of monolithic Ni-based catalysts can be achieved by means of utilizing lower temperatures in each step of production. Alumina-based foams with enhanced properties can be sintered at low temperatures. Metal chlorides can be deposited on the foam surface by aerosol assisted routes and almost completely reduced at very low temperature, 533 K, if modified with addition of only 0.1 wt% of Pd. Desired metal loading is obtained by using different amount of chloride precursor solutions. Ni-Pd/Al_2_O_3_ catalysts with 5 wt% and 20 wt% of Ni were prepared by using chloride precursors for Ni and Pd. After reduction at 533 K, these catalysts had similar microstructures to Ni/Al_2_O_3_ catalysts with the same metal content, which were obtained by reduction of chloride precursor at 633 K. Reduction degree of Ni-Pd/Al_2_O_3_ catalysts reached a value of 99.4 wt% and 98.2 wt% for 5 and 20 wt% Ni loading, respectively.

## Figures and Tables

**Table 1:** Reaction mechanisms and the corresponding equations.

*f*(*α*)	Equations	Reaction mechanism
*D*_1_	*α* ^2^ = *k* · *t*	One-dimensional diffusion
*D*_2_	(1 − *α*)ln⁡(1 − *α*) + *α* = *k* · *t*	Two-dimensional diffusion (cylindrical symmetry)
*D*_3_	[1 − (1 − *α*)^1/3^]^2^ = k · t	Three-dimensional diffusion (spherical symmetry, Jander equation)
*D*_4_	(1 − 2/3*α*) − (1 − *α*)^2/3^ = *k* · *t*	Three-dimensional diffusion (spherical symmetry, Ginstling-Braunshtein equation)
*F*_1_	−ln⁡(1 − *α*) = *k* · *t*	First-order reaction (unimolecular decay law)
*A*_2_	[−ln⁡(1 − *α*)]^1/2^ = *k* · *t*	Random nucleation (Avrami I equation)
*A*_3_	[−ln⁡(1 − *α*)]^1/3^ = *k* · *t*	Random nucleation (Avrami II equation)
*R*_2_	1 − (1 − *α*)^1/2^ = *k* · *t*	Phase-boundary controlled (cylindrical symmetry)
*R*_3_	1 − (1 − *α*)^1/3^ = *k* · *t*	Phase-boundary controlled (spherical symmetry)

## References

[1] Firouzdor V., Sridharan K., Cao G., Anderson M., Allen T. R. (2013). Corrosion of a stainless steel and nickel-based alloys in high temperature supercritical carbon dioxide environment. *Corrosion Science*.

[2] Jõeleht M., Pirso J., Juhani K., Viljus M., Traksmaa R. (2014). The formation of reactive sintered (Ti, Mo)C-Ni cermet from nanocrystalline powders. *International Journal of Refractory Metals and Hard Materials*.

[3] Ma J., Qin M., Zhang L., Zhang R., Qu X. (2013). Microstructure and magnetic properties of Fe-50%Ni alloy fabricated by powder injection molding. *Journal of Magnetism and Magnetic Materials*.

[4] Meng X.-F., Li D.-H., Shen X.-Q., Liu W. (2010). Preparation and magnetic properties of nano-Ni coated cenosphere composites. *Applied Surface Science*.

[5] Li X. D., Elkedim O., Nowak M., Jurczyk M., Chassagnon R. (2013). Structural characterization and electrochemical hydrogen storage properties of Ti_2-x_Zr_x_Ni (*x* = 0, 0.1, 0.2) alloys prepared by mechanical alloying. *International Journal of Hydrogen Energy*.

[6] Basri S., Kamarudin S. K., Daud W. R. W., Yaakob Z., Kadhum A. A. H. (2014). Novel anode catalyst for direct methanol fuel cells. *The Scientific World Journal*.

[7] Plascencia G., Utigard T. (2009). The reduction of Tokyo and Sinter 75 nickel oxides with hydrogen. *Chemical Engineering Science*.

[8] Otomo J., Furumoto Y., Hatano H., Hatanaka T., Oshima Y. (2013). Nickel oxide redox processes with oxide ion conductor-supported nickel oxide in dry and humidified methane: effect of oxide ion conductors on induction period in nickel oxide reduction and subsequent hydrogen production. *Fuel*.

[9] Delmon B. (1969). *Introduction à la Cinétique Hétérogène*.

[10] Richardson J. T., Scates R., Twigg M. V. (2003). X-ray diffraction study of nickel oxide reduction by hydrogen. *Applied Catalysis A: General*.

[11] Chatterjee R., Banerjee S., Banerjee S., Ghosh D. (2012). Reduction of nickel oxide powder and pellet by hydrogen. *Transactions of the Indian Institute of Metals*.

[12] Utigard T. A., Wu M., Plascencia G., Marin T. (2005). Reduction kinetics of Goro nickel oxide using hydrogen. *Chemical Engineering Science*.

[13] Janković B. (2007). Isothermal reduction kinetics of nickel oxide using hydrogen: conventional and Weibull kinetic analysis. *Journal of Physics and Chemistry of Solids*.

[14] Piotrowski K., Mondal K., Lorethova H., Stonawski L., Szymański T., Wiltowski T. (2005). Effect of gas composition on the kinetics of iron oxide reduction in a hydrogen production process. *International Journal of Hydrogen Energy*.

[15] Go K. S., Son S. R., Kim S. D. (2008). Reaction kinetics of reduction and oxidation of metal oxides for hydrogen production. *International Journal of Hydrogen Energy*.

[16] Suh Y. J., Jang H. D., Chang H. K., Hwang D. W., Kim H. C. (2005). Kinetics of gas phase reduction of nickel chloride in preparation for nickel nanoparticles. *Materials Research Bulletin*.

[17] Stopić S. R., Ilić I. E., Uskoković O. P. (1997). Effect of Pd, Cu, and Ni additions on the kinetics of NiCl_2_ reduction by hydrogen. *Metallurgical and Materials Transactions B: Process Metallurgy and Materials Processing Science*.

[18] Stopić S., Friedrich B., Gürmen S. Synthesis of particles of Ni- and Co-powders by ultrasonic spray of NiCl_2_ and Co(NO_3_)_2_ and hydrogen reduction pyrolysis.

[19] Gurmen S., Ebin B., Stopić S., Friedrich B. (2009). Nanocrystalline spherical iron-nickel (Fe-Ni) alloy particles prepared by ultrasonic spray pyrolysis and hydrogen reduction (USP-HR). *Journal of Alloys and Compounds*.

[20] Pérez-Hernández R., Gutiérrez-Martínez A., Palacios J., Vega-Hernández M., Rodríguez-Lugo V. (2011). Hydrogen production by oxidative steam reforming of methanol over Ni/CeO_2_-ZrO_2_ catalysts. *International Journal of Hydrogen Energy*.

[21] Kamberović Ž., Sokić M., Matković V., Andić Z., Korać M., Nikolić V. (2012). Effects of additives on nickel (II)-chloride hydrogen reduction for production of nanocomposite catalysts. *Metalurgia International*.

[22] Juan-Juan J., Román-Martínez M. C., Illán-Gómez M. J. (2009). Nickel catalyst activation in the carbon dioxide reforming of methane: effect of pretreatments. *Applied Catalysis A: General*.

[23] Profeti L. P. R., Dias J. A. C., Assaf J. M., Assaf E. M. (2009). Hydrogen production by steam reforming of ethanol over Ni-based catalysts promoted with noble metals. *Journal of Power Sources*.

[24] Yoshida K., Okumura K., Miyao T. (2008). Oxidative steam reforming of methane over Ni/*α*-Al_2_O_3_ modified with trace Pd. *Applied Catalysis A: General*.

[25] Ciambelli P., Palma V., Palo E. (2010). Comparison of ceramic honeycomb monolith and foam as Ni catalyst carrier for methane autothermal reforming. *Catalysis Today*.

[26] Nikolić V., Kamberović Ž., Anđić Z., Korać M., Sokić M. (2014). Synthesis of *α*-Al_2_O_3_ based foams with improved properties as catalyst carriers. *Materials and Technology*.

[27] Nikolić V., Kamberović Ž., Anđić Z., Korać M., Sokić M., Maksimović V. (2014). Influences of synthesis methods and modifier addition on the properties of Ni-based catalysts supported on reticulated ceramic foams. *International Journal of Minerals, Metallurgy, and Materials*.

[28] Sharp H. J., Brindley W. G., Achar N. B. N. (1966). Numerical data for some commonly used solid state reaction equations. *Journal of the American Ceramic Society*.

